# Renal Survival in Children with Glomerulonephritis with Crescents: A Pediatric Nephrology Research Consortium Cohort Study

**DOI:** 10.3390/jcm9082385

**Published:** 2020-07-26

**Authors:** Joseph G. Maliakkal, M. John Hicks, Mini Michael, David T. Selewski, Katherine Twombley, Michelle N. Rheault, Meredith Seamon, Jason M. Misurac, Cheryl L. Tran, Loretta Reyes, Joseph T. Flynn, Ali M. Onder, Alexandru R. Constantinescu, Vaishali Singh, Cynthia Pan, Abiodun Omoloja, Qiang Wu, William E. Smoyer, Guillermo Hidalgo, Scott E. Wenderfer

**Affiliations:** 1 Pediatric Nephrology, Baylor College of Medicine and Texas Children’s Hospital, Houston, TX 77030, USA; mxmichae@texaschildrens.org; 2 Pediatric Nephrology, Saint Louis University, St. Louis, MO 63103, USA; 3 Pathology, Baylor College of Medicine, Texas Children’s Hospital, Houston, TX 77030, USA; mjhicks@texaschildrenshospital.org; 4 Pediatric Nephrology, Medical University of South Carolina, Charleston, SC 29425, USA; selewski@musc.edu (D.T.S.); twombley@musc.edu (K.T.); 5 Pediatric Nephrology, University of Minnesota, Minneapolis, MN 55455, USA; rheau002@umn.edu; 6 Pediatric Nephrology, The University of Utah, Salt Lake City, UT 84112, USA; Meredith.Seamon@hsc.utah.edu; 7 Pediatric Nephrology, University of Iowa, Iowa City, IA 52242, USA; jason-misurac@uiowa.edu; 8 Pediatric Nephrology, Mayo Clinic, Rochester, MN 55902, USA; Tran.Cheryl2@mayo.edu; 9 Pediatric Nephrology, Emory University, Atlanta, GA 30322, USA; loretta.reyes@emory.edu; 10 Pediatric Nephrology, University of Washington, Seattle, WA 98115, USA; joseph.flynn@seattlechildrens.org; 11 Pediatric Nephrology, University of West Virginia, Morgantown, WV 26506, USA; alim_md@hotmail.com; 12 Pediatric Nephrology, University of Mississippi, Jackson, MS 38677, USA; 13 Pediatric Nephrology, Joe DiMaggio Children’s Hospital, Hollywood, FL 33021, USA; AConstantinescu@mhs.net; 14 Pediatric Nephrology, Medical College of Wisconsin, Milwaukee, WI 53226, USA; vsingh@chw.org (V.S.); cpan@mcw.edu (C.P.); 15 Pediatric Nephrology, Wright State University, Dayton, OH 45435, USA; OmolojaA@childrensdayton.org; 16 Biostatistics, East Carolina University, Greenville, NC 27858, USA; WUQ@ecu.edu; 17 Pediatric Nephrology, The Ohio State University and Nationwide Children’s Hospital, Columbus, OH 43205, USA; william.smoyer@nationwidechildrens.org; 18 Pediatric Nephrology, East Carolina University, Greenville, NC 27858, USA; 19 Pediatric Nephrology, Hackensack Meridian Health, Neptune, NJ 07753, USA

**Keywords:** glomerulonephritis, targeted biologic therapies, immunosuppression, cellular crescents, fibrous crescents

## Abstract

There is no evidence-based definition for diagnosing crescentic glomerulonephritis. The prognostic implications of crescentic lesions on kidney biopsy have not been quantified. Our objective was to determine risk factors for end-stage kidney disease (ESKD) in patients with glomerulonephritis and crescents on kidney biopsy. A query of the Pediatric Nephrology Research Consortium’s Pediatric Glomerulonephritis with Crescents registry identified 305 patients from 15 centers. A retrospective cohort study was performed with ESKD as the primary outcome. Median age at biopsy was 11 years (range 1–21). The percentage of crescents was 3–100% (median 20%). Etiologies included IgA nephropathy (23%), lupus (21%), IgA vasculitis (19%) and ANCA-associated GN (13%), post-infectious GN (5%), and anti-glomerular basement membrane disease (3%). The prevalence of ESKD was 12% at one year and 16% at last follow-up (median = 3 years, range 1–11). Median time to ESKD was 100 days. Risk factors for ESKD included %crescents, presence of fibrous crescents, estimated GFR, and hypertension at biopsy. For each 1% increase in %crescents, there was a 3% decrease in log odds of 1-year renal survival (*p* = 0.003) and a 2% decrease in log odds of renal survival at last follow-up (*p* < 0.001). These findings provide an evidence base for enrollment criteria for crescentic glomerulonephritis in future clinical trials.

## 1. Introduction

In patients with glomerulonephritis (GN), kidney histopathology may include extra-capillary glomerular cell proliferation in the form of a crescent [1,2]. The pathologic diagnosis of “crescentic glomerulonephritis” lacks an evidence-based definition. The pathophysiology may differ by etiology. In some kidney diseases, cellular crescents form in response to circulating or de novo immune complexes. In other diseases, pauci-immune mechanisms predominate. The composition of cellular crescents can include inflammatory as well as parenchymal glomerular cells. In some patients, crescent formation evolves equally in all glomeruli, whereas in other cases smaller numbers of glomeruli become involved in a piecemeal manner over time. Cellular crescents are thought to represent “active” lesions whereas fibrous crescents are considered “chronic” lesions. Although cellular crescents are thought to be more treatable than fibrous crescents, there are few repeat biopsy studies to assess the natural history of crescentic involvement or their reversibility with treatment.

Although many previous studies included patients with ≥50% crescents on biopsy [3,4,5,6,7], inclusion criteria for others range from ≥20% [8,9] to ≥75% [10]. Small cohorts of adult populations have been reported; however, sparse literature exists on outcomes of pediatric GN with crescents [3,4,5,6,7,9,10,11,12,13]. Notably, etiologies of GN differ between children and adults [1,2,3,14,15,16], with post-infectious and immune complex GN occurring more frequently in children and anti-glomerular basement membrane (GBM) and anti-neutrophil cytoplasmic antibody (ANCA) associated-GN occurring more commonly in adults.

Recent years have seen advances in our understanding of GN pathogenesis, including the roles of complement mutations in C3 glomerulopathy [17] and anti-PLA2R antibodies in membranous GN [18], which have led to revised disease classification. Availability of new therapies, including rituximab [19,20] and eculizumab [21] have improved patient outcomes. In one adult study, one-year renal survival improved from 70% in the 1990s to 80% [22]. However, clinical course and outcomes can vary widely: without large cohorts, the effects of clinical variables at time of biopsy have been inadequately studied.

The rarity of the disease, particularly in children, has limited the size of prior cohorts. The largest pediatric trials included 54 and 60 children from single centers in Canada [11] and Germany [13]. The last multi-center pediatric study evaluating 50 children with GN and crescents was published 30 years ago by the Southwest Pediatric Nephrology Study Group (SPNSG) [3]. Renal outcomes were variable, with a 10% rate of end-stage kidney disease (ESKD) in the German cohort (38% median percentage of glomeruli with crescents) and 51% rate in the SPNSG American cohort (where all cases had >50% crescents) [3,13]. Review of a diverse and multicenter contemporary cohort of children with GN with crescents offers the opportunity to describe contemporary outcomes and more importantly to begin development of evidence-based approaches for risk prediction and treatment.

The investigators in the Pediatric Nephrology Research Consortium (PNRC), formerly known as the Midwest Pediatric Nephrology Consortium (MWPNC), assembled a multi-center cohort of pediatric patients with these findings on biopsy since January 2004. The registry includes children with GN and more than one crescent on kidney biopsy. As of May 2019, 15 participating centers have enrolled 342 children into the registry. In this study, we used this registry to identify risk factors associated with ESKD in children with GN with crescents.

## 2. Material and Methods

The PNRC’s Pediatric Glomerulonephritis with Crescents Registry is a multi-center retrospective cohort of prevalent pediatric patients with crescents on kidney biopsy, established in August 2015. The investigations were carried out following the revised rules of the Declaration of Helsinki 2013. IRB approval was obtained at the study-coordinating center, East Carolina University (UMCIRB15-000362, approved 6/17/2015) and locally at each participating center. Nationwide Children’s Hospital maintains the study-specific database using the OpenClinica platform. Enrollment included all eligible subjects identified in administrative and billing databases by text search and ICD-9/10 codes. Eligible subjects were <21 years old [23] at time of native kidney biopsies performed between January 2004 and February 2016. Inclusion criteria required the finding of more than one crescent, as defined by local renal pathologists, and ≥12 months follow-up at the same institution.

The population included in this study comes from a query of the OpenClinica database performed in October 2019 (raw data available upon request). Data were extracted on demographics (age at time of biopsy, self-reported gender, race, and ethnicity) and clinical findings at time of biopsy including height, weight, gross hematuria, microscopic hematuria, proteinuria, and serum creatinine. Hypertension was determined based on blood pressure >95th percentile for age/gender/height [24] or use of antihypertensive medications. Abnormal proteinuria was defined as protein excretion on ≥2 occasions by either dipstick ≥1+, spot urine protein-creatinine ratio >0.2 mg/mg or >8 mg/m^2^/hour on 24 h collection. Biopsy reports were queried for cellular or fibrous crescents (fibrocellular crescents were combined with cellular crescents in the registry) [25]. Etiologies of GN were indicated by site investigators based on all clinical and pathologic data.

**Figure 1:**
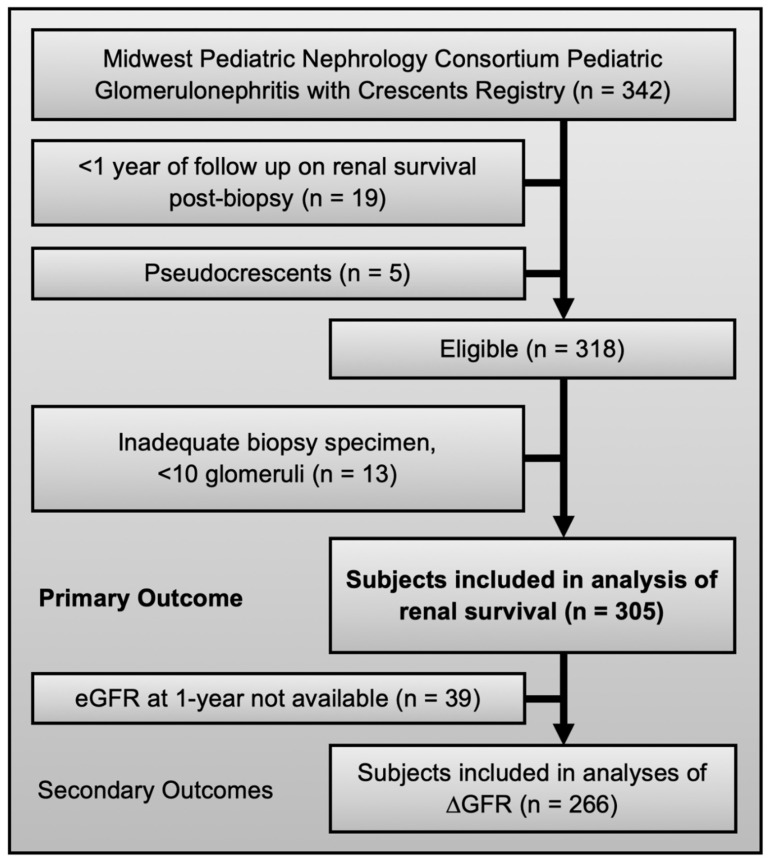
Eligibility and inclusion criteria for the registry and this study.

The primary outcome measure was ESKD, defined as initiation on chronic dialysis (>3 months) or pre-emptive kidney transplantation, regardless of eGFR. Kidney function was determined by calculating eGFR, using the revised Schwartz equation [26]. GFR slope was used to assess changes in kidney function over time [27]. Outcomes were assessed at two time points: 1-year post biopsy (±3 months) and last follow-up. For time-to-event analyses and additional outcome measures, time to ESKD declaration was calculated. The available number of subjects did not allow for full subset analysis, such as outcomes in specific etiologies or after specific therapies. Of 342 subjects enrolled in the registry, 305 had renal survival data and 266 had eGFR data at 1-year (±3 months) follow-up (Figure 1). No missing data were imputed. Sensitivity analysis suggested no acquisition bias due to missing data (Table S6).

Statistical analysis was performed using SAS: Release 9.4. Univariate analysis identified four covariates associated with ESKD at 1-year (percent crescents, presence of fibrous crescent, hypertension at biopsy, and eGFR at biopsy). A logistic regression incorporating these covariates was used to model probability of ESKD. To assess accuracy of models in predicting 1-year outcome, area under the curve (AUC) was measured using receiver operating curve (ROC) analysis. To develop a bedside prediction tool, the threshold of percentage crescents associated with ESKD at 1-year with the highest Youden’s index was identified [28]. Strength of association between continuous numerical data (percent crescents, eGFR, and GFR slope) was assessed using Pearson product moment correlation.

For survival analysis, a Cox proportional hazard model was used to assess time to ESKD or to last follow-up. Since Youden’s index for predicting ESKD at 1-year was 43% crescents, we also compared the survival time to ESKD between patients with ≥43% and <43% crescents.

## 3. Results

The study population included 305 patients from 15 participating pediatric nephrology centers across the United States (Figure 1). The most common etiologies of GN included IgA nephropathy (23%), lupus nephritis (21%), IgA vasculitis (19%), and ANCA-associated GN (13%). Diseases known to present with crescents on kidney biopsy were also seen: idiopathic immune complex GN (6%), pauci-immune GN (4%), and GBM disease (3%). Female subjects comprised 58% of the cohort and mean age at time of biopsy was 11.3 ± 4.3 years (Table 1). Overall, at time of biopsy 45% were hypertensive, 91% had proteinuria, 23% had nephrotic syndrome, 2% had isolated gross hematuria, and 4% had oliguria/anuria. The mean eGFR at biopsy was 72 ± 47 mL/min/1.73 m^2^. Renal replacement therapy was provided in 12% of subjects at time of biopsy (81% intermittent hemodialysis and 19% CRRT).

**Figure 2:**
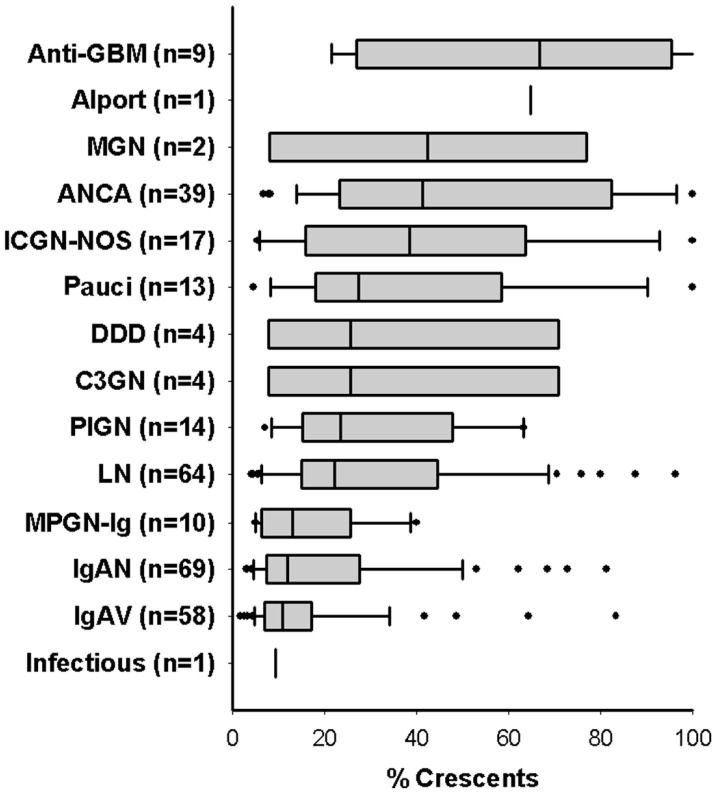
Percent crescents in children with glomerulonephritis and crescents, stratified by etiology. Median percentage of glomerular crescents are shown for all 305 children in the study. Boxes and whiskers represent IQR with 5th and 95th percentiles. Etiologies: anti-glomerular basement membrane disease (anti-GBM), membranous GN (MGN), anti-neutrophil cytoplasmic antibody associated nephritis (ANCA), immune complex GN not otherwise specified (ICGN-NOS), dense deposit disease (DDD), C3 glomerulopathy (C3GN), post-infectious GN (PIGN), lupus nephritis (LN), Ig-predominant membranoproliferative GN (MPGN-Ig), IgA nephropathy (IgAN), IgA renal vasculitis (IgAV).

The median interval between the date of renal onset and the date of biopsy was 26 days (IQR 6–69). The median number of glomeruli assessed was 29 (IQR 20–40) and the median percent of glomeruli with crescents was 20% (IQR 11–41%). Figure 2 shows median percent crescents by etiology. Cellular and fibrous crescents were identified in 92% and 32% of subjects respectively. Cellular and fibrous crescents were seen together in 27% of subjects, compared to 65% of biopsies with only cellular and 4% with only fibrous crescents. When present, the median percentage of glomeruli with cellular crescents was 14% (IQR 7–30%) and the median percentage of fibrous crescents was 9% (IQR 5–17%). There was no co-linearity between percent cellular and percent fibrous crescents. Glomerular endocapillary neutrophilic infiltrates were noted in 47% of biopsies; tubular atrophy, in 53%; interstitial fibrosis, in 61%; and focal necrotizing glomerular lesions, in 31% of cases. Necrotizing lesions were 50% more common in biopsies with glomerular neutrophilic infiltrates. Global glomerulosclerosis was noted in 38% of cases, and 5% had global sclerosis in >50% of the total number of glomeruli. Immunofluorescence (IF) and electron microscopy findings of immune deposition were consistent with etiologies diagnosed.

Following kidney biopsy, 67% of patients were prescribed IV pulse steroids, 44% cyclophosphamide (oral or IV), 15% rituximab, and 9% plasmapheresis. Oral therapy included mycophenolate mofetil (45%) and azathioprine (19%). Therapies provided by specific etiology are shown in Table S1. Therapies provided, stratified by percent crescents, are shown in Table S2.

At 1-year, the prevalence of ESKD was 12%. The prevalence of ESKD, stratified by etiology, is shown in Table S3. Rates of ESKD for IgA vasculitis (n = 58) were 0%, but >50% for idiopathic immune complex GN (n = 17) and anti-GBM disease (n = 9). Patients who developed ESKD at 1-year had a higher mean percent crescents (66% compared to 25% without ESKD, *p* < 0.001), higher prevalence of fibrous crescents (53%, *p* = 0.005), glomerulosclerosis (60%, *p* = 0.02), more hypertension (79%, *p* < 0.001), and lower eGFR at time of biopsy (21 mL/min/1.73 m^2^, *p* < 0.001) (Table 1). Using multiple logistic regression, higher percentage of crescents, presence of fibrous crescents, presence of hypertension, and decreased eGFR at time of biopsy each independently predicted ESKD at 1-year (Table 2). For each 1% increase in percent crescents, the log odds of progression to ESKD increased by 3% (95% C.I. 1–5%), and for each 1 mL/min/1.73 m^2^ decrease in eGFR, the log odds of ESKD increased by 4% (95% C.I. 2–6%). The predicted probability of ESKD at 1-year is calculated by

(1) $$\frac{\exp\left(-1.884+0.032*\%crescents+0.610*Fb+0.693*HT-0.039*eGFR\right)}{1+\exp\left(-1.884+0.032*\%crescents+0.610*Fb+0.693*HT-0.039*eGFR\right)}$$

Percentage of crescents also correlated inversely with eGFR at biopsy (R = −0.48, *p* < 0.001) and the eGFR at 1-year (R = −0.49, *p* < 0.001). When stratified by etiology, this correlation was strongest in children with ANCA-GN (Table S4). For children with GN, stratification by percent crescents distinguished 1-year renal outcomes (Figure S1A). In the four most common etiologies in the cohort (lupus, IgA nephropathy, IgA vasculitis, and ANCA-GN), the eGFR at time of biopsy differed substantially (Figure S2), with the most severe renal failure in children with ANCA-GN and crescents. Over the first year post biopsy, the trajectory of eGFR improved in all top etiologies of GN, although to varying degrees (Figure S3). One-year renal outcomes in children with post-infectious GN and C3GN with crescents were superior to others in the cohort, including those with immune complex GN and dense deposit disease.

**Figure 3:**
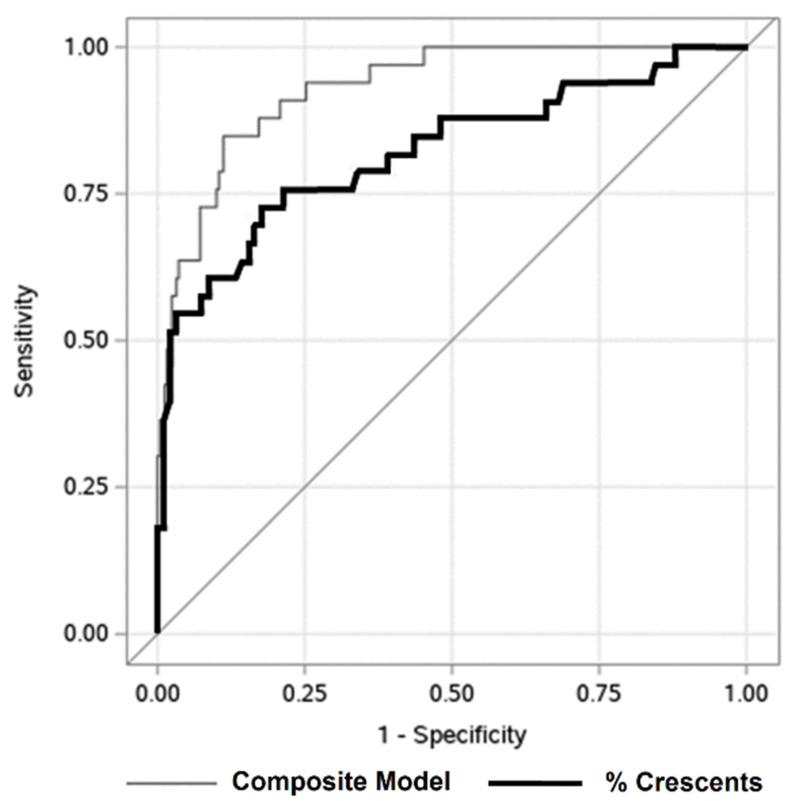
Predicting 1-year renal survival in children with glomerulonephritis with crescents. Receiver operating characteristic curve describing association of percentage of glomerular crescents at biopsy (black line, area under the curve = 0.82) and composite model (gray line, area under the curve = 0.93) with ESKD at one year post biopsy. The AUC for the composite model is significantly higher (*p*-value = 0.001). Under the composite model, a predicted probability of 0.14 is the optimal cutoff. When only percent crescents are used to predict end-stage kidney disease (ESKD) at 1-year, 43% is the optimal cutoff.

A composite equation, incorporating the four independent covariates identified on multiple logistic regression, was significantly associated with ESKD at 1-year (AUC 0.93) (Figure 3). The AUC was 0.82 for percent crescents alone. The maximal Youden’s index (maximal combination of sensitivity, 74%, and specificity, 83%) was measured at a threshold of 43% crescents. There were 72 subjects with ≥43% crescents (Table S5). The mean age, 13 years, was higher than in patients with <43% crescents (11 years). Patients with ≥43% crescents had more hypertension (56 vs. 41%) and lower eGFR at biopsy (32 vs. 84 mL/1.73 m^2^/min). For children with GN, applying this threshold for percent crescents distinguished both eGFR at biopsy and at 1-year (Figure S1B). Patients with ≥43% crescents tended to have worse outcomes regardless of etiology (Figure S4). This threshold also distinguished better between patients deemed higher risk by their treating nephrologist, based on higher utilization of oral immunosuppression (like MMF) and less use of more intensive therapy (Table S2).

Median follow-up was 2.8 years (range 1–11.6). ESKD at last follow-up was 16%. Median time to ESKD was 100 days (range 0–9.8 years). Although percent crescents were associated with long-term renal survival (*p* < 0.001), there was no correlation between percent crescents and time to ESKD (R = 0.19, *p* = 0.2). Long-term renal survival for specific etiologies and specific therapies tended to mirror 1-year survival (Table S3). The 1-year eGFR predicted eGFR at last follow-up (R = 0.36, *p* < 0.001). Among the more common etiologies represented, percent crescents correlated with eGFR in ANCA-GN and IgA nephropathy (Table S4). Using Cox proportional hazard analysis, higher percentage of crescents, presence of fibrous crescents, presence of hypertension at biopsy, and decreased eGFR at biopsy each independently predicted time to ESKD with censoring at last follow-up (Table 2). For each 1% increase in the percentage of crescents, the log hazard of progression to ESKD increased by 2% (95% C.I. 1–4%), and for each 1 mL/min/1.73 m^2^ decrease in eGFR at biopsy, the log hazard of ESKD increased by 3% (95% C.I. 1–4%). The overall hazard of ESKD is proportional to

(2) exp(0.019*%crescents+1.09*Fb+0.804*HT−0.029*eGFR)

Since length of follow-up varied widely for subjects in the registry, the acute GFR slope (from biopsy to 1-year post biopsy) was compared to total GFR slope (from biopsy to last follow-up, Figures S4 and S5). Children with lupus or post-infectious GN and crescents had more improvement and children with IgA nephropathy and crescents had less improvement after the first year. However, for some etiologies the acute and total GFR slope differed substantially. Initial improvements in kidney function in children with IgA vasculitis and immune complex GN tended to show decline over the long term.

**Figure 4:**
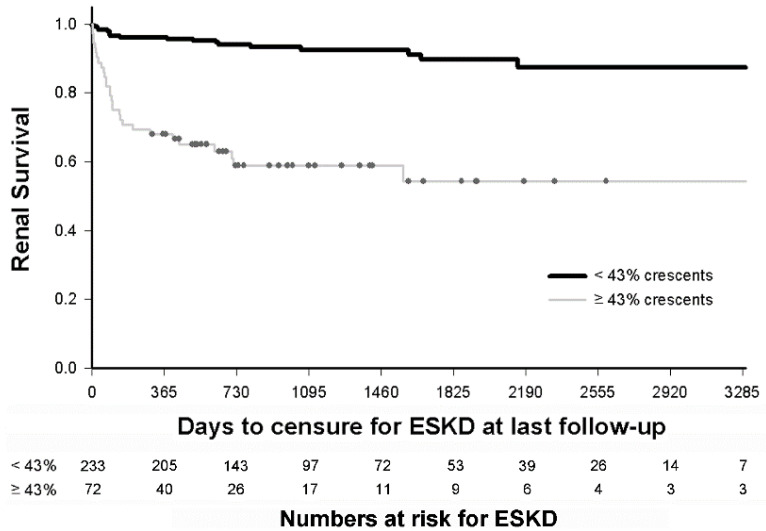
Predicting long-term renal survival in children with glomerulonephritis with crescents. Kaplan Meier survival analysis demonstrates that children with glomerulonephritis and ≥43% crescents (n = 72) have decreased survival than children with <43% crescents (n = 233), at a median of 3 years follow up (hazard ratio of 6.77, 95% CI: 3.76–12.2). The numbers in each subset remaining at risk after each year of follow-up are indicated.

We applied the threshold of 43% crescents to determine its ability to identify patients at risk for poor long-term outcomes. Kaplan Meier survival analysis demonstrated that presence of ≥43% crescents on initial biopsy was associated with decreased renal survival (log rank test *p* < 0.0001) (Figure 4).

## 4. Discussion

This analysis of a large multi-center pediatric cohort of children with glomerulonephritis with crescents provides the strongest evidence to date that the percentage of glomerular crescents associates with renal outcomes. Findings validate the 30-year-old SPNSG pediatric study [3] and more recent single center studies [6,13], where percent crescents associated with ESKD. Previous pediatric studies that failed to identify a significant association were underpowered or perhaps misappropriated high-risk subjects based on too stringent a definition for crescentic GN.

This study extends to children the findings from adults. In a multicenter cohort of 1118 adult patients with IgA nephropathy and crescents, ≥25% crescents associated with a composite outcome (ESKD or ≥50% decline in eGFR) with a hazard ratio of 2.29 [8]. This evidence prompted a revision of the Oxford Classification of IgA nephropathy to add C0 (no crescents), C1 (<25% crescents), or C2 (≥25% crescents). Studies of adult lupus patients have culminated in the use of 25% and 50% thresholds for both cellular and fibrous crescents in pathologic indices [25,29,30,31]. In 172 and 406 adults with lupus nephritis and crescents, each 1% increase in crescents corresponded to 2–4% increased risk of ESKD or doubling of serum creatinine [32,33]. Unfortunately, however, similar studies of 40, 78, and 101 pediatric patients with IgA vasculitis and GN with crescents failed to validate the proposed International Study of Kidney Disease in Children (ISKDC) classification thresholds for crescents, due to small sample sizes [34,35,36].

ESKD at 1-year is a clinically important short-term outcome measure in patients with GN [5,6,13,14,22,24,37,38,39]. More than half of children with GN and crescents who progress to ESKD do so within the first year after initial biopsy [5,6,13]. A U.K. study reported rates of ESKD of 53% at 9.5 years, compared to 40% after 1-year [5]. A study from India reported ESKD rates of 19% at one and 36% at 6 years [6]. Several adult studies of GN with crescents also report renal survival at 1-year as an outcome measure [14,22,24,37,38,39]. Although the rate of ESKD in our cohort (12%) was lower than the U.K. and Indian studies (that enrolled only patients with >50% crescents on biopsy), the rate was 35% in our more comparable subset with >43% crescents. The median time to ESKD in our population was 100 days, confirming that 1-year renal survival is an important outcome measure for use in future trials of pediatric GN with crescents.

Based on our modeling, percent crescents were one of four useful predictors of outcome at the time of biopsy. Findings are consistent with univariate analyses in a cohort of 60 German children [13]. As linear measures, the contribution of overall increased risk was comparable for eGFR and percent glomeruli with crescents. As discrete variables, presence of hypertension and fibrous crescents also contributed to similar degrees. Our composite risk equation may be useful in protocol development for future pediatric clinical trials of GN.

We also report a bedside estimate of ESKD risk using a threshold of 43% glomerular crescents. Applying the higher threshold of 50% crescents often quoted in textbooks (and used in the SPNSG study [3]) has decreased sensitivity in our cohort (65% versus 74%). Conversely, applying the lower threshold of 20% crescents, previously used by Alsaad et al. [9], has decreased specificity in our cohort (53% versus 83%). In clinical situations, pediatric nephrologists may find this evidence-based risk estimate more practical than the composite risk equation. However, due to the linear relationship between percent crescents and renal outcome, our study does not support use of crescentic GN as a clinically useful diagnosis. Rather, future studies of glomerular crescents should focus on the lesion as a prognostic biomarker and include all percentages of glomerular involvement.

Limitations of this study include its retrospective design and possibility of recall and selection bias. Systematic review of local pathology databases, when available, reduced the magnitude of this bias. The registry relies upon local pathologists to identify and characterize crescentic lesions, rather than using central pathology. To mitigate this concern, biopsy reports were reviewed by a single experienced renal pathologist (MJH) to resolve ambiguities noted by investigators. More precise consensus definitions for cellular, fibrocellular, and fibrous crescents have now been published for patients with lupus nephritis [40], but were unavailable at time of subject enrollment in the PNRC registry. To minimize the possibility of sampling error at kidney biopsy, cases with <10 glomeruli were excluded. The interval between the date of renal onset and the date of kidney biopsy was not included in our risk assessment, but was relatively short. The PNRC registry does not capture the time between the date of biopsy and the start of treatment. However, reducing both of these intervals are known to improve renal outcomes [13]. Although the median follow-up time was only 2.8 years, 45 subjects had over 6 years (2190 days) of follow-up and 10 patients had over 9 years (3285 days, Figure 4). The relatively small sample sizes of the specific etiologies of GN results in insufficient statistical power to assess the contribution of etiology in our regression modeling. Finally, the relatively small number of ESKD events limited the number of covariates that could be included in regression modeling.

Our study also has several notable strengths. We investigated the largest cohort to date of pediatric patients with GN and crescents, including 72 subjects with ≥43% crescents and 62 subjects with ≥50% crescents. This is also the first multi-center study to include pediatric patients with all percentages of crescentic involvement. Inclusion across the full spectrum of GN with crescentic involvement created the unique opportunity to systematically investigate crescents as a prognostic biomarker across the entire spectrum of glomerular diseases. Furthermore, given the broad geographic distribution of participating centers, and inclusive demographic spectrum of the study population, the findings of our study are likely generalizable to the entire US pediatric population.

## 5. Conclusions

There is a linear relationship between the percent crescents at initial biopsy in pediatric GN and the risk for ESKD. Together with eGFR, presence of hypertension, and presence of any fibrous crescents on biopsy, percent crescents should become even more important for risk stratification in future clinical trials. Although it is assumed that etiology of GN also predicts risk of poor outcomes, larger study populations are needed to understand the risk associated with each etiology in children. The Pediatric Glomerulonephritis with Crescents Registry offers the opportunity for more questions to be interrogated as additional sites and patients are enrolled.

## Figures and Tables

**Table 1:** Characteristics of children with crescents on kidney biopsy with renal survival compared to those with end-stage kidney disease (ESKD) at one year post biopsy.

		N	Overall (N = 305)	No ESKD at 1-Year (N = 270)	ESKD at 1-Year (N = 35)
**Age, mean (sd), years**		305	11.3 (4.3)	11.1 (4.3)	12.7 (4.4)
**Gender female, N (%)**		305	176 (58)	56%	65%
**Race, N (%)**	**Caucasian**	285	168 (59)	54%	62%
	**African American**		43 (15)	13%	21%
	**Hispanic**		45 (16)	15%	12%
	**Asian**		18 (6)	5%	3%
	**Other**		11 (4)	6%	0
**Hypertension at Biopsy, N (%)**		295	133 (45)	40%	79%
**Proteinuria at Biopsy, N (%)**		305	278 (91)	92%	86%
**eGFR at Biopsy, mean (sd), mL/min/1.73 m^2^**		290	72 (47)	79 (45)	21 (20)
**Glomerular crescents, mean (sd), percentage**		305	30 (26)	25 (21)	66 (33)
**Presence of Cellular Crescents, N (%)**		301	277 (92)	92%	94%
**Presence of Fibrous Crescent(s), N (%)**		302	97 (32)	29%	53%
**Presence of Glomerulosclerosis, N (%)**		305	116 (38)	35%	60%

**Table 2:** Regression analysis of the association between clinical and histologic findings at the time of biopsy and ESKD at one year and last follow-up.

**ESKD at One Year**					
**Covariates**	**Parameter**	**SE**	**Odds Ratio**	**95% CI**	***p*-Value**
Percent Crescents (per 1%)	0.029	0.010	1.03	1.01–1.05	0.003
Presence of Fibrous Crescents (Yes/No)	0.646	0.255	3.64	1.34–9.89	0.011
Hypertension at Biopsy (Yes/No)	0.715	0.283	4.18	1.38–12.7	0.011
eGFR at Biopsy (per mL/min/1.73 m^2^)	−0.043	0.012	0.96	0.94–0.98	0.001
**ESKD at last follow-up**					
**Covariates**	**Parameter**	**SD**	**Hazard Ratio**	**95% CI**	***p*-Value**
Percent Crescents (per 1%)	0.019	0.006	1.02	1.01–1.04	<0.001
Presence of Fibrous Crescents (Yes/No)	1.09	0.309	2.90	1.58–5.29	<0.001
Hypertension at Biopsy (Yes/No)	0.804	0.345	2.31	1.18–4.57	0.015
eGFR at Biopsy (per mL/min/1.73 m^2^)	−0.029	0.007	0.97	0.96–0.99	<0.001

## Supplementary Materials

The following are available online at https://www.mdpi.com/2077-0383/9/8/2385/s1, Figure S1: eGFR at biopsy and one year in children with acute glomerulonephritis, Figure S2: eGFR at biopsy and one year in children with the four most common etiologies of glomerulonephritis with crescents, Figure S3: eGFR at biopsy and one year, in the four most common etiologies, stratified by %crescents, Figure S4: Change in eGFR in children with less common etiologies, stratified by %crescents, Figure S5: Change in eGFR in individual children with glomerulonephritis with crescents, stratified by etiology and %crescents, Table S1: Percentage of patients exposed to therapy stratified by etiology, Table S2: Percentage of patients exposed to therapy stratified by percent crescents on initial biopsy, Table S3: Renal survival stratified by etiology of nephritis, Table S4: Correlations between %crescents and eGFR or eGFR slope stratified by disease, Table S5: Characteristics of children with ≥43% crescents on kidney biopsy compared to children with <43% crescents. Table S6: Characteristics of children in the PNRC registry, as of October 2019.
