# Supplementary material for: Renal Survival in Children with Glomerulonephritis with Crescents: A Pediatric Nephrology Research Consortium Cohort Study
Source: J Clin Med. 2020 Jul 26;9(8):2385. doi: 10.3390/jcm9082385 (PMC7464981; doi:10.3390/jcm9082385)
Supplement: Supplementary file 1 [file jcm-09-02385-s001.docx]

jcm-869573-supplementary

Table S1

Percentage of patients exposed to therapy stratified by etiology among all patients included in study (n = 305). Etiologies: anti-neutrophil cytoplasmic antibody associated nephritis (ANCA), IgA nephropathy (IgAN), IgA renal vasculitis (IgAV), post-infectious GN (PIGN), C3 glomerulonephritis (C3GN), anti-glomerular basement membrane disease (anti-GBM), Ig-predominant membranoproliferative GN (MPGN-Ig), immune complex GN not otherwise specified (ICGN-NOS), dense deposit disease (DDD), membranous GN (MGN). Therapies: azathioprine (AZA), cyclophosphamide (IV or oral) (CYC), mycophenolate mofetil (MMF), rituximab (RTX).

| **Etiology** | **N** | **IV Steroids**  N (%) | **CYC**  N (%) | **RTX**  N (%) | **Pheresis**  N (%) | **MMF**  N (%) | **AZA**  N (%) |
| --- | --- | --- | --- | --- | --- | --- | --- |
| Lupus nephritis | 64 | 55 (85%) | 50 (77%) | 13 (23%) | 3 (5%) | 51 (78%) | 5 (9%) |
| ANCA | 39 | 34 (87%) | 32 (82%) | 22 (56%) | 14 (36%) | 11 (28%) | 19 (49%) |
| IgAN | 69 | 25 (36%) | 9 (13%) | 0 | 0 | 30 (43%) | 8 (12%) |
| IgAV | 58 | 28 (48%) | 9 (16%) | 2 (3%) | 0 | 23 (40%) | 19 (33%) |
| PIGN | 14 | 12 (86%) | 4 (29%) | 0 | 0 | 4 (31%) | 0 |
| C3GN | 4 | 1 (25%) | 1 (25%) | 1 (25%) | 2 (50%) | 1 (25%) | 0 |
| Anti-GBM | 9 | 9 (100%) | 8 (89%) | 1 (11%) | 6 (67%) | 1 (11%) | 2 (22%) |
| Pauci-immune | 13 | 13 (100%) | 11 (85%) | 5 (38%) | 0 | 4 (31%) | 4 (31%) |
| MPGN-Ig | 10 | 6 (60%) | 1 (10%) | 0 | 0 | 5 (50%) | 0 |
| ICGN-NOS | 17 | 13 (76%) | 5 (29%) | 1 (6%) | 0 | 6 (35%) | 1 (6%) |
| DDD | 4 | 4 (100%) | 1 (25%) | 1 (25%) | 1 (25%) | 1 (25%) | 0 |
| MGN | 2 | 1 (50%) | 1 (50%) | 1 (50%) | 0 | 1 (50%) | 1 (50%) |
| Staphylococcal GN | 1 | 1 (100%) | 1 (100%) | 0 | 0 | 0 | 0 |
| Alport | 1 | 1 (100%) | 0 | 0 | 0 | 0 | 0 |
| Total | 305 | 203 (67%) | 133 (44%) | 47 (15%) | 26 (9%) | 138 (45%) | 59 (19%) |

# **Table S2**

Percentage of patients exposed to therapy stratified by percent crescents on initial biopsy among all patients included in study (n = 305). Therapies: azathioprine (AZA), cyclophosphamide IV or oral (CYC), mycophenolate mofetil (MMF), rituximab (RTX).

| **% Crescents** | **N** | **IV Steroids**  N (%) | **CYC**  N (%) | **RTX**  N (%) | **Pheresis**  N (%) | **MMF**  N (%) | **AZA**  N (%) |
| --- | --- | --- | --- | --- | --- | --- | --- |
| ≤25% | 186 | 102 (55%) | 55 (30%) | 21 (11%) | 7 (4%) | 84 (45%) | 31 (16%) |
| 25–50% | 62 | 51 (82%) | 35 (56%) | 9 (15%) | 4 (6%) | 33 (53%) | 11 (18%) |
| 50–75% | 27 | 25 (93%) | 18 (67%) | 5 (19%) | 5 (19%) | 10 (37%) | 9 (33%) |
| ≥75% | 30 | 25 (83%) | 25 (83%) | 11 (37%) | 10 (33%) | 10 (33%) | 8 (27%) |
|  |  |  |  |  |  |  |  |
| <43% | 233 | 139 (60%) | 81 (35%) | 30 (13%) | 10 (4%) | 111 (48%) | 39 (17%) |
| ≥43% | 72 | 63 (88%) | 52 (72%) | 16 (22%) | 16 (22%) | 26 (36%) | 20 (29%) |

# Table S3

Renal survival stratified by etiology of nephritis among all patients included in study (n = 305). Etiologies: anti-glomerular basement membrane disease (anti-GBM), immune complex GN not otherwise specified (ICGN-NOS), membranous GN (MGN), dense deposit disease (DDD), anti-neutrophil cytoplasmic antibody associated nephritis (ANCA), post-infectious GN (PIGN), IgA nephropathy (IgAN), Ig-predominant membranoproliferative GN (MPGN-Ig), IgA renal vasculitis (IgAV), C3 glomerulonephritis (C3GN).

| **Etiology** | **N** | **Renal Survival**  **At 1 Year**  N (%) | **Renal Survival**  **At Latest F/U**  N (%) | **Median**  **F/U** (months) | **Median Time**  **To ESKD** (days) |
| --- | --- | --- | --- | --- | --- |
| Alport | 1 | 0 (0%) | 0 (0%) | 15 | 23 |
| Anti-GBM | 9 | 4 (44%) | 3 (33%) | 36 | 99 |
| ICGN-NOS | 17 | 8 (47%) | 7 (41%) | 43 | 89 |
| MGN | 2 | 1 (50%) | 1 (50%) | 24 | 30 |
| DDD | 4 | 2 (50%) | 2 (50%) | 53 | 105 |
| ANCA | 39 | 31 (79%) | 27 (69%) | 30 | 30 |
| Pauci-immune | 13 | 12 (92%) | 12 (92%) | 31 | 58 |
| PIGN | 14 | 13 (93%) | 13 (93%) | 23 | 60 |
| IgAN | 69 | 65 (94%) | 62 (90%) | 35 | 140 |
| Lupus nephritis | 64 | 63 (97%) | 60 (92%) | 36 | 373 |
| MPGN-Ig | 10 | 10 (100%) | 9 (90%) | 45 | 1037 |
| IgAV | 58 | 58 (100%) | 57 (98%) | 31 | 787 |
| Staphylococcal GN | 1 | 1 (100%) | 1 (100%) | 31 | -- |
| C3GN | 4 | 4 (100%) | 4 (100%) | 49 | -- |
| Total | 305 | 271 (89%) | 259 (85%) | 33 | 100 |

# Table S4

Correlations between % crescents and eGFR or eGFR slope (ΔeGFR/Year) among patients with eGFR data stratified by disease (n = 266). *P-value <0.05. Etiologies: membranous GN (MGN), C3 glomerulonephritis (C3GN), dense deposit disease (DDD), anti-neutrophil cytoplasmic antibody associated nephritis (ANCA), immune complex GN not otherwise specified (ICGN-NOS), anti-glomerular basement membrane disease (anti-GBM), IgA renal vasculitis (IgAV), post-infectious GN (PIGN), Ig-predominant membranoproliferative GN (MPGN-Ig).

| **Etiology** | **N** | **R Coefficient with % Crescents** | | | | |
| --- | --- | --- | --- | --- | --- | --- |
|  |  | **eGFR**  **At Biopsy** | **eGFR**  **At 1 Year** | **ΔeGFR/Year**  **Over Year 1** | **eGFR**  **At Last F/U** | **ΔeGFR/Year**  **At Last F/U** |
| MGN | 2 | -0.75 | -0.94 | -0.25 | -0.94 | -0.25 |
| C3GN | 4 | -0.65 | -0.75 | 0.83 | -0.75 | 0.83 |
| DDD | 4 | -0.63 | -0.46 | 0.36 | -0.53 | -0.41 |
| ANCA | **37** | **-0.62*** | **-0.53*** | 0.04 | **-0.43*** | 0.06 |
| ICGN-NOS | 17 | -0.49 | -0.34 | 0.08 | -0.20 | -0.02 |
| IgA nephropathy | **56** | **-0.48*** | -0.03 | 0.06 | **-0.39*** | -**0.27*** |
| Anti-GBM | 9 | -0.47 | -0.38 | -0.13 | -0.54 | -0.51 |
| IgAV GN | **48** | **-0.45*** | -0.07 | 0.27 | -0.18 | **0.41*** |
| PIGN | 13 | -0.36 | -0.47 | 0.09 | -0.30 | 0.19 |
| Lupus nephritis | 55 | -0.33 | -0.11 | 0.26 | -0.10 | 0.30 |
| Pauci-immune | 12 | -0.27 | -0.04 | 0.32 | -0.09 | 0.06 |
| MPGN-Ig | 8 | -0.22 | -0.41 | 0.32 | -0.32 | 0.24 |
| Alport | 1 | -- | -- | -- | -- | -- |
| Total | 266 | **-0.48*** | **-0.49 *** | 0.07 | **-0.36*** | 0.11 |

# Table S5

Characteristics of children with ≥43% crescents on kidney biopsy compared to children with <43% crescents on kidney biopsy.

|  | | **<43% Crescents**  **(N = 233)** | **≥43% Crescents**  **(N = 72)** | **P-value** |
| --- | --- | --- | --- | --- |
| **Age (Years)** | | **10.8** | **12.8** | **<0.001** |
| Gender (% Female) | | 58 | 58 | 0.902 |
| Race (%) | Caucasian | 58 | 61 | 0.250 |
|  | African American | 13 | 21 |  |
|  | Hispanic | 18 | 10 |  |
|  | Asian | 7 | 4 |  |
|  | Other | 4 | 3 |  |
| **Hypertension at Biopsy** | | **41** | **56** | **0.024** |
| Proteinuria at Biopsy | | 92 | 88 | 0.213 |
| **eGFR at Biopsy (mL/min/1.73m^2^)** | | **84** | **32** | **<0.001** |
| Presence of Cellular Crescents | | 92 | 94 | 0.535 |
| Presence of Fibrous Crescents | | 31 | 33 | 0.702 |

# Table S6

Sensitivity anlaysis was performed to test for acquisition bias. Characteristics of children in the PNRC registry, and subset who met eligibility as defined in Figure 1. Also shown are the characteristics of children with available data for studying the primary and secondary outcomes.

|  | | **Enrolled in Registry**  **(N = 342)** | **Eligible (n=318)** | **Primary outcome**  **(N = 305)** | **Secondary outcomes (N = 266)** |
| --- | --- | --- | --- | --- | --- |
| **Age at biopsy, mean (sd), years** | | 11.0 (4.4) | 11.0 (4.3) | 11.3 (4.3) | 11.5 (4.3) |
| **Gender female, N (%)** | | 191 (56) | 179 (56) | 176 (58) | 157 (59) |
| **Race, N (%)** | **Caucasian** | 189 (55) | 175 (55) | 168 (55) | 151 (57) |
|  | **African American** | 44 (13) | 43 (14) | 43 (14) | 35 (13) |
|  | **Hispanic** | 51 (15) | 48 (15) | 45 (15) | 44 (17) |
|  | **Asian** | 20 (6) | 19 (6) | 18 (6) | 16 (6) |
|  | **Other / Unknown** | 39 (11) | 32 (10) | 31 (10) | 19 (7) |
| **Hypertension at biopsy, N (%)** | | 147 (43) | 136 (43) | 133 (45) | 119 (45) |
| **eGFR at biopsy, mean (sd), mL/min/1.73m^2^** | | 68 (50) | 71 (46) | 72 (47) | 69 (47) |
| **Glomerular crescents, mean (sd), percentage** | | 30 (26) | 30 (26) | 30 (26) | 31 (26) |
| **Presence of cellular crescents, N (%)** | | 310 (91) | 290 (92) | 277 (92) | 250 (94) |
| **Presence of fibrous crescent(s), N (%)** | | 106 (31) | 101 (32) | 97 (32) | 90 (34) |
| **Presence of global glomerulosclerosis, N (%)** | | 126 (37) | 118 (37) | 116 (38) | 108 (41) |
| **Anti-GBM, N (%)** | | 9 (3) | 9 (3) | 9 (3) | 9 (3) |
| **Alport, N (%)** | | 1 (0.3) | 1 (0.3) | 1 (0.3) | 1 (0.4) |
| **Membranous GN, N (%)** | | 4 (1.1) | 3 (0.9) | 2 (0.6) | 2 (0.8) |
| **ANCA GN, N (%)** | | 41 (12) | 39 (12) | 39 (13) | 37 (14) |
| **ICGN-NOS, N (%)** | | 22 (6) | 21 (7) | 17 (6) | 17 (6) |
| **Pauci-immune GN, N (%)** | | 16 (5) | 14 (4) | 13 (4) | 12 (5) |
| **DDD, N (%)** | | 6 (2) | 5 (2) | 4 (1) | 4 (2) |
| **C3GN, N (%)** | | 7 (2) | 6 (2) | 4 (1) | 4 (2) |
| **Post Infectious GN, N (%)** | | 17 (5) | 15 (5) | 14 (5) | 13 (5) |
| **Lupus nephritis, N (%)** | | 68 (20) | 65 (20) | 64 (21) | 55 (21) |
| **MPGN-Ig, N (%)** | | 10 (3) | 10 (3) | 10 (3) | 8 (3) |
| **IgAN, N (%)** | | 75 (22) | 70 (22) | 69 (23) | 56 (21) |
| **IgAV, N (%)** | | 64 (19) | 61 (18) | 58 (19) | 48 (18) |
| **Infectious, N (%)** | | 1 (0.3) | 1 (0.3) | 1 (0.3) | 0 |
| **IV steroids, N (%)** | | 229 (67) | 212 (67) | 203 (67) | 185 (70) |
| **Cyclophosphamide (PO or IV), N (%)** | | 152 (44) | 143 (45) | 133 (44) | 123 (46) |
| **Rituximab, N (%)** | | 54 (16) | 51 (16) | 47 (15) | 45 (17) |
| **Pheresis, N (%)** | | 30 (9) | 28 (9) | 26 (9) | 26 (10) |
| **Mycophenolate mofetil, N (%)** | | 147 (43) | 141 (44) | 138 (45) | 126 (47) |
| **Azathioprine, N (%)** | | 62 (18) | 60 (19) | 59 (19) | 53 (20) |
| **< 43% crescents, N (%)** | | 258 (75) | 241 (76) | 233 (76) | 198 (74) |
| **≥ 43% crescents, N (%)** | | 84 (25) | 77 (24) | 72 (24) | 68 (26) |

| **A** | **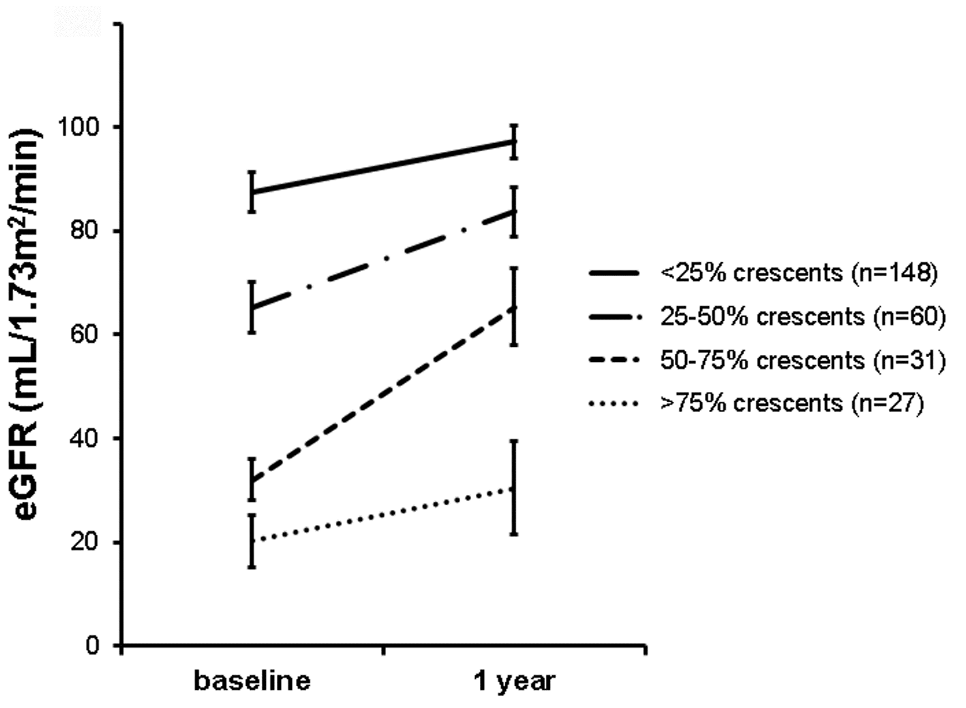** |
| --- | --- |
| **B** | 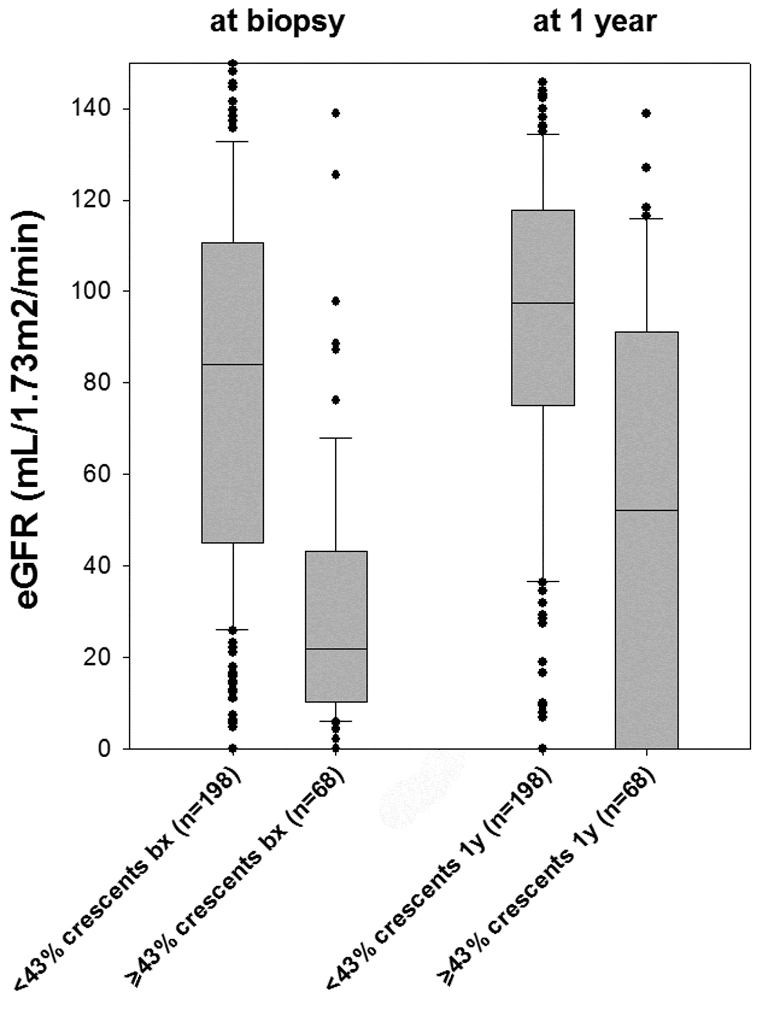 |

**Figure S1: (A)** Change in eGFR over first year after biopsy in children with acute glomerulonephritis of any etiology, stratified by percentage of glomerular crescents on biopsy. Shown are median values with error bars indicating the standard error. **(B)** Estimated GFR at biopsy and one year after biopsy, in children with acute glomerulonephritis of any etiology, stratified by percentage of glomerular crescents on biopsy. Cutoff of 43% glomeruli with crescents was selected based on evidence from univariate analysis shown in Figures 4 and 5. Numbers of subjects per group indicated (n = 266 total). Boxes and whiskers represent medians, IQR, 5th and 95th percentiles.


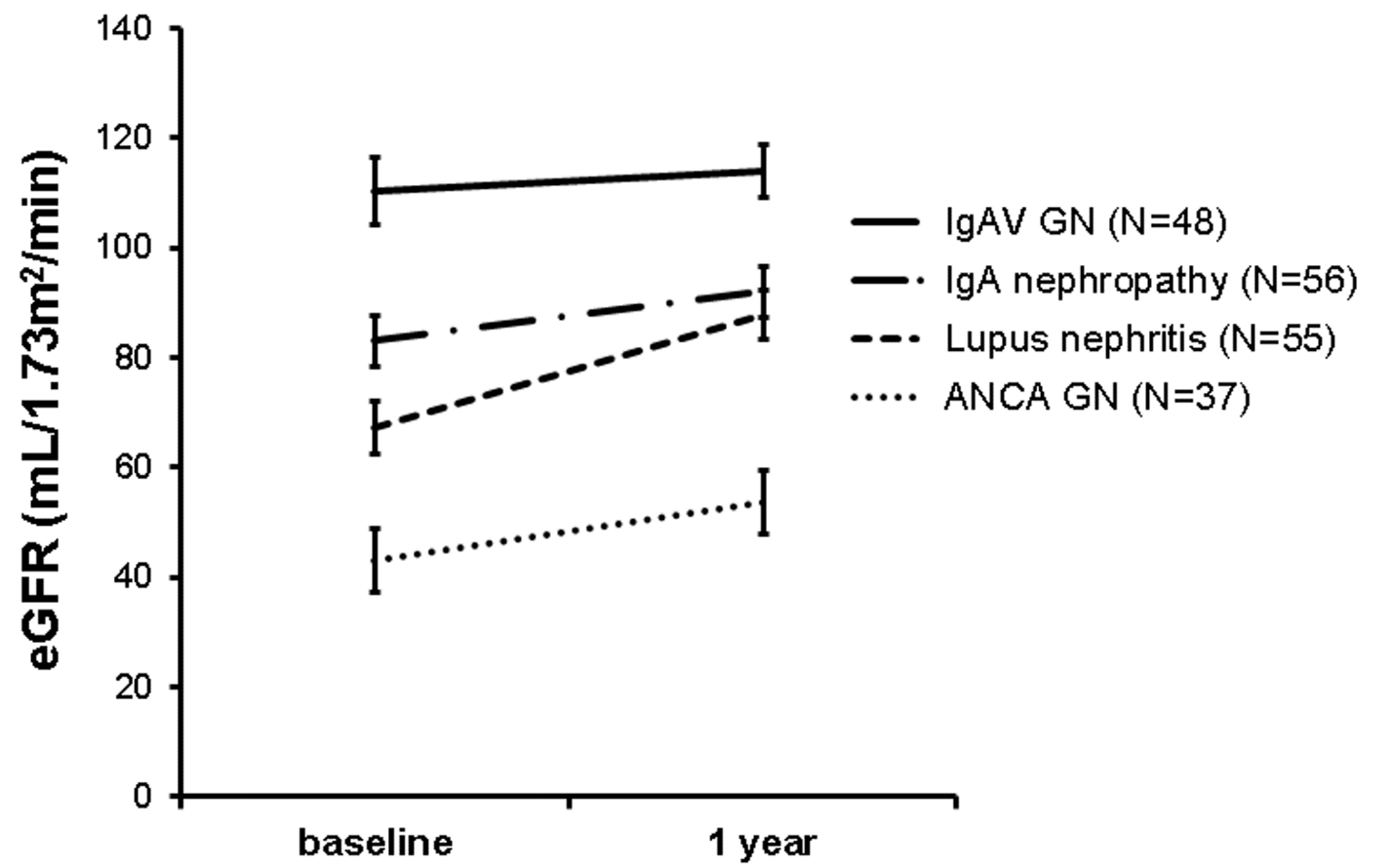


**Figure S2:** Estimated GFR at biopsy and one year after biopsy, in children with the four most common etiologies of glomerulonephritis with crescents, regardless of the percentage of glomerular crescents on biopsy or the treatments received. Numbers of subjects per group indicated in legend. Shown are mean values with error bars indicating the standard error.

**
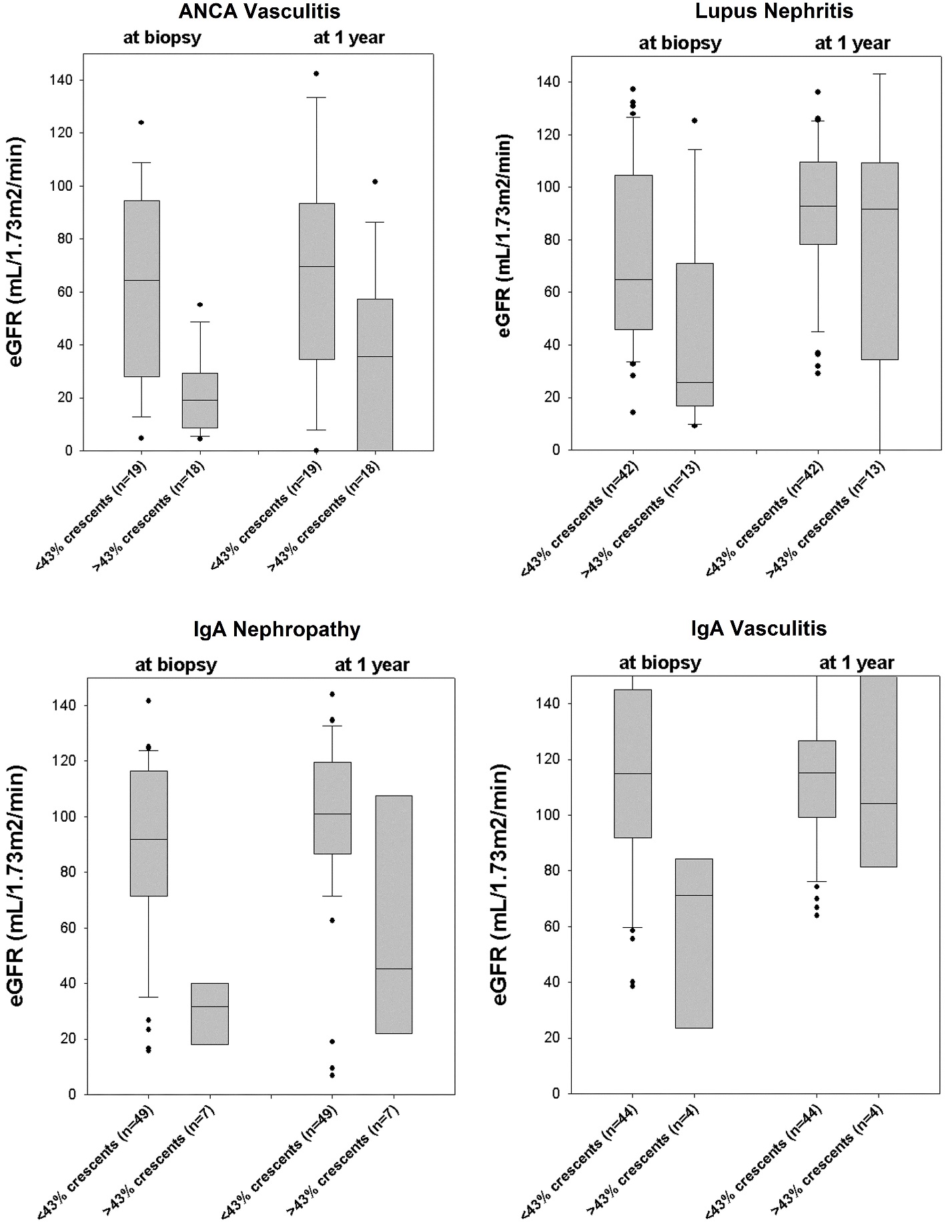
**

**Figure S3:** Estimated GFR at biopsy and one year after biopsy, in children with the four most common etiologies of glomerulonephritis with crescents, stratified by percentage of glomerular crescents on initial biopsy, regardless of treatments received. Cutoff of 43% glomeruli with crescents was selected based on evidence from univariate analysis shown in Figures 4 and 5. Numbers of subjects per group indicated. Boxes and whiskers represent medians, IQR, 5th and 95th percentiles. Anti-neutrophil cytoplasmic antibody (ANCA).


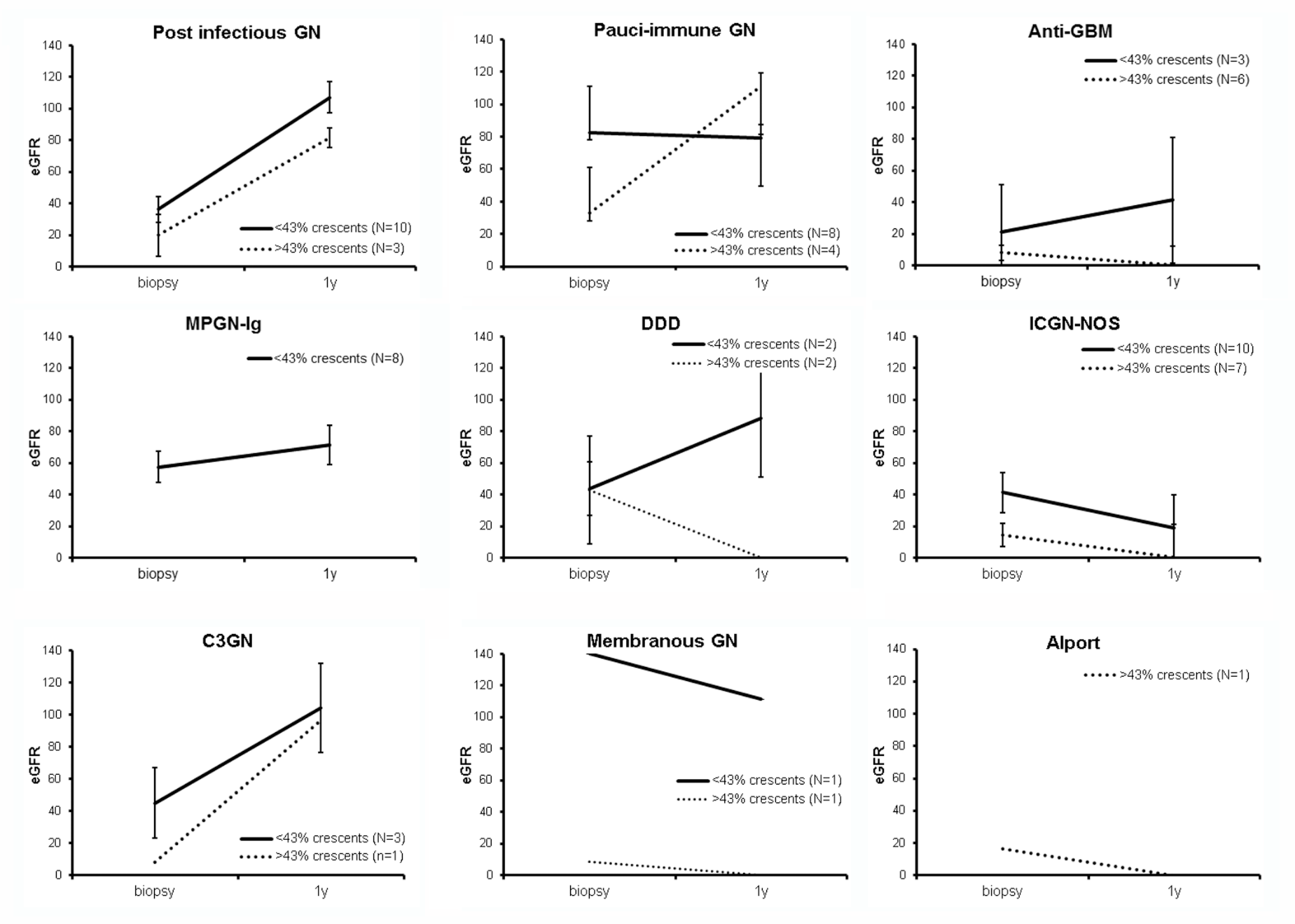


**Figure S4:** Change in eGFR in children with less common etiologies of glomerulonephritis and crescents, stratified by the percentage of glomerular crescents detected on biopsy. Optimal cutoff of 43% glomeruli with crescents based on evidence in Figures 4 and 5. Numbers of subjects per group indicated in legend. Shown are median values, with error bars indicating standard errors.

**Supplemental Figure 5 (continued)**

**Figure S5:** Change in eGFR in individual children with glomerulonephritis with crescents, stratified by etiology and percentage of glomerular crescents on initial biopsy, regardless of treatments received. Shown are values for each subject with ≥43% crescents (dotted lines) and <43% crescents (solid lines) on initial biopsy.
